# The Influence of Modularity on Cranial Morphological Disparity in Carnivora and Primates (Mammalia)

**DOI:** 10.1371/journal.pone.0009517

**Published:** 2010-03-03

**Authors:** Anjali Goswami, P. David Polly

**Affiliations:** 1 Department of Genetics, Evolution and Environment and Department of Earth Sciences, University College London, London, United Kingdom; 2 Department of Geological Sciences, Indiana University, Bloomington, Indiana, United States of America; Michigan State University, United States of America

## Abstract

**Background:**

Although variation provides the raw material for natural selection and evolution, few empirical data exist about the factors controlling morphological variation. Because developmental constraints on variation are expected to act by influencing trait correlations, studies of modularity offer promising approaches that quantify and summarize patterns of trait relationships. Modules, highly-correlated and semi-autonomous sets of traits, are observed at many levels of biological organization, from genes to colonies. The evolutionary significance of modularity is considerable, with potential effects including constraining the variation of individual traits, circumventing pleiotropy and canalization, and facilitating the transformation of functional structures. Despite these important consequences, there has been little empirical study of how modularity influences morphological evolution on a macroevolutionary scale. Here, we conduct the first morphometric analysis of modularity and disparity in two clades of placental mammals, Primates and Carnivora, and test if trait integration within modules *constrains* or *facilitates* morphological evolution.

**Principal Findings:**

We used both randomization methods and direct comparisons of landmark variance to compare disparity in the six cranial modules identified in previous studies. The cranial base, a highly-integrated module, showed significantly low disparity in Primates and low landmark variance in both Primates and Carnivora. The vault, zygomatic-pterygoid and orbit modules, characterized by low trait integration, displayed significantly high disparity within Carnivora. 14 of 24 results from analyses of disparity show no significant relationship between module integration and morphological disparity. Of the ten significant or marginally significant results, eight support the hypothesis that integration within modules constrains morphological evolution in the placental skull. Only the molar module, a highly-integrated and functionally important module, showed significantly high disparity in Carnivora, in support of the facilitation hypothesis.

**Conclusions:**

This analysis of within-module disparity suggested that strong integration of traits had little influence on morphological evolution over large time scales. However, where significant results were found, the primary effect of strong integration of traits was to constrain morphological variation. Thus, within Primates and Carnivora, there was some support for the hypothesis that integration of traits within cranial modules limits morphological evolution, presumably by limiting the variation of individual traits.

## Introduction

The correlated evolution of traits, whether due to genetic, developmental, or functional interactions, has been a rich source of study for decades. Early researchers identified “functional components” to systems such as the cranium [1], and subsequent studies of morphological integration [2], [3] and phenotypic modularity [4] have sought to quantify and generalize these relationships among morphological traits. Perhaps the first quantitative examination of phenotypic trait relationships can be attributed to Olson and Miller [2], expounded in their book *Morphological Integration* [3]. Olson and Miller's argument was a simple one: many trait changes that occur during the course of evolution do not occur independently of each other. More specifically, traits that are related by proximity in development or function have greater influence on each other than on more distant traits.

Measurements of trait variation and covariation, the tendency for traits to vary in a coordinated manner, have shown that there are significant differences in the relationships among traits and, in some cases, have demonstrated that some traits are linked by strong correlations, while others show little or no correlation [4], [5]. By examining the networks of these relationships, one can identify modules, sets of highly-correlated traits that have only weak correlations with traits outside of the module. Thus modules are recognized by two aspects: 1) autonomy from other modules or traits; and 2) strong integration of traits within the module.

For morphologists and paleontologists, this emergence of studies of phenotypic modularity is particularly important, because the quantitative methods used to identify modularity can be applied equally to living, extinct or rare taxa. Moreover, the concept of evolutionary modularity is tied to the genotype-phenotype map, with most studies of modularity and integration having focused on the relationships among modules observed in genetic, developmental, and morphological systems [6]–[35]. The broad range of studies demonstrates that modularity can be applied to diverse systems [4], [30] and can be observed in morphology through quantitative analysis, making it a useful concept for integrative studies of evolutionary morphology. Modularity has also been tied to some of the most fundamental and interesting questions in morphological evolution, including evolvability and constraints on morphological variation, the generation of novelties, and the production of morphological diversity [11], [35]–[42].

Modularity is thought to affect evolution in several ways. When considering the evolution of a single module, the most important feature is the integration of within-module traits. Strong correlations among traits potentially limit the variation of any individual trait [28], which may ultimately slow the rate of evolution or constrain the morphological evolution of the module to a smaller range of possible variability. Alternatively, the same correlations may coordinate and perhaps accelerate evolution of a module [43], which, assuming that environmental conditions and selection pressures are comparable, would be expected to result in more rapid and thus greater diffusion through possible morphospace.

Modularity itself also evolves, and new modularity may arise by the parcellation or fragmentation of one large group of traits into smaller groups by the severing of interactions bridging the two new groups [40], [41]. The new modules can then vary independently of each other, possibly increasing the system's “evolvability”, its potential for morphological variation and evolution. Such a mechanism of dissociation of parts has been suggested to be a process that counteracts developmental canalization and genetic pleiotropy, which would otherwise increase unchecked over evolutionary time and severely curb the generation of variation [41]. On the other hand, modularity may arise by traits becoming more integrated over evolutionary time, perhaps due to new functional associations, to create new or larger modules [40], [41]. Thus, the evolution of modularity is best studied by considering modules in the context of other putatively independent modules, because the evolutionary effects of modularization will be manifested in two ways within a single biological system: within modules (integration) and across modules (autonomy).

In this regard, there have been several studies conducted on the relationship between overall cranial shape and variation in an individual module, primarily on basicranial interactions in hominids [44]–[48]. However, these studies haven't addressed the question of whether the level of integration, specifically high correlations among traits, actually constrains variation within a module. Without testing of these hypotheses about modularity's influence on morphology, it is impossible to accurately assess the evolutionary significance of modularity or of observed differences in modularity across taxa that demonstrate that modularity itself evolves [32], [49].

**Figure 1:**
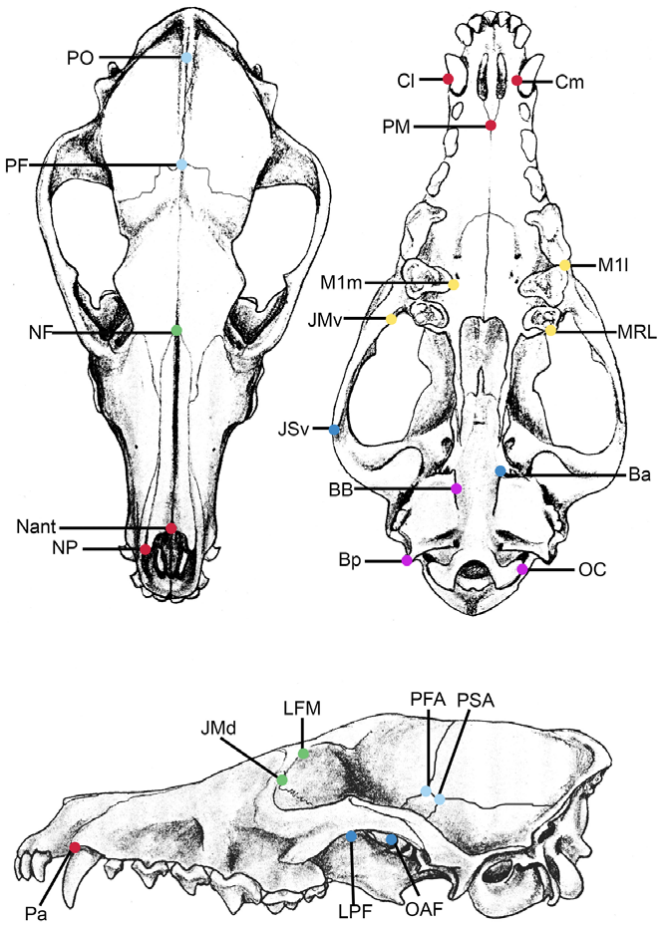
Landmarks and module associations used in analyses, shown on *Vulpes* vulpes. The six different colors used to mark landmarks correspond to six cranial modules: anterior oral-nasal (red); molar (yellow); orbit (green); zygomatic-pterygoid (dark blue); vault (light blue); and basicranium (purple). Symmetrical landmarks are shown on one side only. Labels are as in Table S2. Modified from Gilbert [66].

A recent study demonstrated that patterns of cranial phenotypic modularity are strongly conserved across placental mammals [32]. Morphometric analyses of 3-D cranial landmarks, using cluster analyses of landmark covariances, followed by Fisher's z-transformation and Student's t-test to determine if grouped landmarks displayed significantly higher covariances than observed between groups, identified six sets of traits that were consistently recovered in the examined species (Fig. 1). Correlations between traits that were not in the same cluster (i.e. correlations between traits in different modules) were consistently zero or not significantly different from zero (Table A2 in [32]). While all of the six groups of traits fulfilled a practical definition of phenotypic modularity, in having significantly stronger correlations within the module than across modules in at least some taxa, three modules, the orbit, zygomatic-pterygoid, and cranial, were significantly correlated in less than half of the taxa. Mean within-module correlations, averaged across all species sampled in [32], and 95% confidence intervals from bootstrap analyses (1000 replicates) for each clade are detailed in Table 1. Based on these analyses, we subdivided these six modules into “strong” modules (anterior oral-nasal, molar, and basicranial) and “weak” modules (orbit, zygomatic-pterygoid, and vault).

**Table 1:** Modules analysed in disparity analyses.

Module	% Taxa	# Landmarks	Mean r	Upper CI	Lower CI
**Carnivora**					
Anterior Oral-Nasal	95%	10	0.72	0.77	0.66
Basicranium	92%	6	0.64	0.69	0.60
Molar	77%	7	0.46	0.51	0.42
Cranial Vault	48%	6	0.38	0.44	0.33
Orbit	48%	5	0.38	0.43	0.32
Zygomatic-Pterygoid	15%	8	0.25	0.30	0.20
**Primates**					
Anterior Oral-Nasal	87%	10	0.57	0.62	0.52
Basicranium	58%	6	0.43	0.49	0.36
Molar	61%	7	0.41	0.46	0.36
Cranial Vault	18%	6	0.23	0.30	0.18
Orbit	5%	5	0.24	0.28	0.20
Zygomatic-Pterygoid	5%	8	0.17	0.21	0.13

% Taxa refers to the number of taxa in which all the traits in a module were significantly correlated. Within-module trait correlations and significance values for each species are detailed in Table A2 of [32], but, as an approximate guide, r = .41 is significant at the p  = .05 level for the average species sample size. Mean r and Upper and Lower CI refers to the mean correlation and 95% confidence intervals among all of the traits in a module, averaged across all species in each clade, as determined by bootstrap analysis (1000 replicates).

Here, we use these observed differences in the cranial modules to address one aspect of modularity's influence on morphological evolution: how the magnitude of trait integration influences the disparity of individual modules. Disparity measures the morphological divergence among taxa and is a measure of the variety of organisms, rather than simply their numbers [50]. In the context of this paper, the traits of interest are morphometric landmarks, and disparity is measured with partial Procrustes distances between each specimen and the mean shape for a module. We measure the morphological disparity of each module across 77 placental mammal species from the orders Primates and Carnivora, the same taxa used in the original analyses of cranial modularity. If modularity influences morphological evolution, one would expect differences in the morphological disparity of the “strong modules”, those with significant within-module trait correlations in most taxa, and the “weak modules”, those with mean within-module trait correlations that are significant in some examined species, but not significant in most of the taxa in each clade.

**Figure 2:**
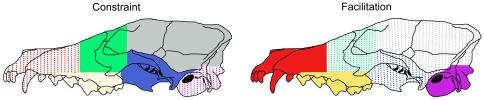
Models for constraint and facilitation hypotheses. Disparity expectations for each of the six cranial modules under the two models of constraint and facilitation. Solid shading indicates significantly high disparity/landmark variance; stippled shading indicates significantly low disparity/landmark variance. Regions of solid and stippled shading approximate the areas bounded by the landmarks shown in Fig. 1.

We test two specific models that represent the extremes of the possible ways that modularity may influence morphological evolution: *constraint* and *facilitation*. As shown in Figure 2, if modularity constrains morphological evolution, then the high correlations among traits in the “strong” modules should limit the variation of individual traits, resulting in low morphological disparity (*constraint*). Correspondingly, the low correlations in the “weak” modules should exert little control on the variation of individual traits, allowing high morphological disparity to evolve. If it is instead correct that modularity promotes morphological evolution via coordinated transformations of sets of traits (*facilitation*), then the high correlations in the “strong” modules should promote trait variation, resulting in high morphological disparity, while “weak” modules should show low disparity (Fig. 2). Of course, there are many intermediate possibilities. Different modules may in fact show different patterns, with some supporting constraint and others facilitation. Nor does the relationship between modularity and disparity need to be linear; perhaps only the strongest modules exert any influence on disparity, and most correlations have no effect at all. However, without a baseline, it is difficult to establish the boundaries of the manifold intermediate hypotheses, and thus this study, in representing the first attempt to test for a relationship between modularity and disparity, focuses on only the two simplest models of facilitation and constraint.

## Results

**Figure 3:**
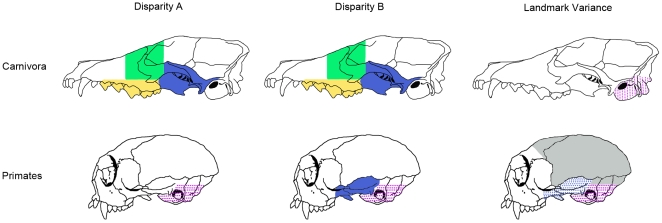
Results of disparity analyses. Disparity observations for the six cranial modules for each of the three analyses described in Methods: disparity A, disparity B, and landmark variance. Solid shading indicates significantly high disparity or variance; stippled shading indicates significantly low disparity or variance. As in Fig. 2, regions of solid and stippled shading only approximate the areas covered by the landmarks shown in Fig. 1. Results are generally congruent across the three measures, and, of the significant results, the majority support the constraint model, depicted in Fig. 2.

In analyses of Carnivora, where random sets were drawn from the full set of landmarks (Disparity A), the orbit and zygomatic-pterygoid modules showed significantly high disparities (both with p = .01), but the molar group also displayed marginally significantly high disparity (p = .03; Table 2, Fig. 3). When Primates was analysed, again with random sets drawn from the full landmark set (Disparity A), no module showed significantly high disparity, but the basicranial module had marginally significantly lower disparity (p = .03) than observed in random sets of landmarks. That only two of the 12 analyses of Disparity A resulted in significant results, and two additional analyses were marginally significant, suggests that in most cases, module integration does not have a strong influence on module disparity. However, of these four results, three supported the hypothesis that module integration constrains disparity, while only one marginally significant result, for the molar module, supported the facilitation hypothesis.

**Table 2:** Predictions and significant results for disparity analyses.

Module	Constraint Model	Facilitation Model	Carnivora Disparity A	Carnivora Disparity B	Primates Disparity A	Primates Disparity B
Anterior Oral-Nasal	−	+				
Basicranium	−	+			−	−
Molar	−	+	+	+		
Cranial Vault	+	−		+		
Orbit	+	−	+	+		
Zygomatic-Pterygoid	+	−	+	+		+

+ indicates higher disparity than random sets;–indicates lower disparity than random sets. No other results are significant at the p<0.05 level. Constraint Model and Facilitation Facilitation are the hypothetical models being tested for the effects of module integration on morphological evolution, as described in the text. Disparity A and B are described in Materials and Methods.

In analyses where disparity from one of the three “strong” modules was compared to a distribution generated from random sets of landmarks drawn only from a combined set of “weak” modules, and vice versa (Disparity B), results were generally concordant with those for Disparity A (Table 2, Fig. 3). In Carnivora, the molar group had marginally significantly higher disparity than the random sets of “weak” module landmarks (p = 0.05), while the orbit, zygomatic-pterygoid, and vault groups all had significantly or marginally significantly higher disparity than the random sets of “strong” module landmarks (p<0.001, p<0.001, and p = 0.03, respectively). In Primates, the basicranial group had significantly lower disparity than the random sets of “weak” module landmarks (p = 0.002), while the zygomatic-pterygoid group had a significantly higher disparity than the random sets of “strong” modules (p<0.001). Thus out of twelve analyses of Disparity B, four were significant, two marginally significant, and five of these supported the constraint hypothesis.

In the last series of analyses, direct comparisons of landmark variances of each module from a single Procrustes superimposition of all cranial landmarks, Carnivora showed marginally significant differences in landmark variance across modules (Kruskal-Wallis test, p = 0.02), while Primates showed extremely significant differences across modules (p<0.001). In Primates, Mann-Whitney tests showed that this reflects primarily the significantly low landmark variances in the basicranial region and high landmark variances in the vault (Table 3, Fig. 3). In this analysis, the zygomatic-pterygoid region appears to have relatively low landmark variance, in contrast to the previous analyses. In Carnivora, Mann-Whitney tests primarily involved the low landmark variance of the basicranial region (Table 3, Fig. 3). Neither Carnivora nor Primates showed significant differences between bins of all “strong” and all “weak” landmarks (p = .1 and p = .53, respectively).

**Table 3:** Comparisons of module landmark variances.

	Anterior Oral-Nasal	Basicranium	Molar	Vault	Orbit	Zygomatic-Pterygoid
**Anterior Oral-Nasal**		0.09	1	0.87	1	0.56
**Basicranium**	***0.02***		1	0.08	***0.05***	1
**Molar**	1	1		0.68	1	1
**Vault**	1	***0.04***	1		***0.05***	<**0.01**
**Orbit**	0.86	***0.02***	1	1		0.30
**Zygomatic-Pterygoid**	1	1	1	1	1	

Bonferroni-corrected p-values from Mann-Whitney tests of differences in landmark variances among modules. Carnivora is in the lower triangle, Primates in the upper triangle. Bold indicates significance at the p<0.01 level, bold italics indicate marginal significance (0.05>p>0.01).

## Discussion

The constraint hypothesis predicts that “strong” modules, sets of highly-correlated traits, should display low morphological disparity. In contrast, the facilitation hypothesis predicts that strong modules show display high morphological disparity. Likewise, regions of the skull with little to no integration of traits in most taxa (“weak” modules) should, or more conservatively, could display high disparity under the constraint hypothesis or low disparity under the facilitation hypothesis. Within these two clades of placental mammals, there was some support for the hypothesis that integration of landmarks within cranial modules limits morphological evolution, presumably by limiting the variation of individual landmarks, but most results did not support a significant relationship between integration and morphological disparity.

The orbit, zygomatic-pterygoid, and cranial vault are the most weakly-integrated regions of the cranium, with low mean within-module correlations in both Carnivora and Primates and little to no correlation among landmarks in many individual species. In Carnivora, these three “weak” modules displayed significantly higher disparity than that of randomly-generated sets of traits in five out of six analyses (Table 2, Disparity A and B). In Primates, only the zygomatic-pterygoid region was significantly more disparate than random sets of landmarks (Disparity B). Thus, all of the significant results for the “weak” modules are consistent with the constraint hypothesis.

For the “strong” modules, the anterior oral-nasal, basicranium, and molar modules, only four of the 12 analyses of Disparity A and B returned significant results. Contrary to the constraint hypothesis, the molar module displayed significantly higher disparity than random sets of landmarks for both Disparity A and B in Carnivora. The basicranium in Primates was the only highly-integrated module showing lower disparity, which was also reflected in analyses of landmark variance from a single Procrustes superimposition, in both Primates and Carnivora. However, it is worth noting that the basicranial module is significant in only a small majority of primate species, as reflected in the bootstrap analyses of mean within-module correlation across species (Table 1).

Thus, out of 24 total analyses, ten analyses returned significant results and eight of these support the constraint hypothesis. Of course, there is no reason why landmark integration only constrains or only facilitates morphological evolution; a mixed pattern is certainly possible. However, it is important to note that 14 out of the 24 analyses supported neither the constraint nor the facilitation hypothesis. The lack of significant results could reflect either the combination of facilitation and constraint counteracting each other over macroevolutionary time scales, or the lack of a consistently strong relationship between integration and morphological evolution.

The molar module is the primary example of a strongly integrated set of landmarks that also displays high disparity. Of course, teeth are well recognized for their evolutionary disparity, stemming from their crucial functional importance in obtaining and processing food. The high disparity of the molar region is perhaps unsurprising in Carnivora, which is one of the most ecologically diverse mammalian clades. Among the carnivoran species sampled in this study are hypercarnivores, frugivores, folivores, and social insectivores, as well as a variety of omnivorous forms, all of which differ greatly from each other in dental morphology. The high disparity of the molar region may simply reflect strong selective pressure on highly functional traits, which may override the potential constraints of strong integration of traits. The alternative to the constraint hypothesis is the facilitation hypothesis, which suggests that strong integration of traits may actually accelerate morphological evolution. Unfortunately, given that the molar module was the only one of the “strong” modules to display high disparity, it is difficult to determine if integration actually facilitated evolution in this module.

It has been argued that the basicranium is a relatively conservative region of the cranium, under relatively low selection pressure [51], and the analyses reported here demonstrate the low disparity of the basicranium in support this view. However, the rostral region, which includes the anterior dentition, could be argued to be under similar selective pressure as the molar region, both for feeding and display purposes. This module has the strongest correlations of any region of the cranium, yet returned no significant results in any analysis. This region may present a worthwhile area for future studies incorporating additional taxa, as it clearly serves important functional purposes but also shows much strong correlations than observed in the molar region.

It is also worth noting that there were several differences between Primates and Carnivora, possibly reflecting ecological and evolutionary differences among these clades. While both clades showed low disparity (Primates) or low landmark variance (Primates and Carnivora) in the basicranial region, and high disparity (Carnivora) or high landmark variance (Primates and Carnivora) in the vault regions, Primates did not show high disparity in the molar regions. As noted above, this difference in the disparity of the molar region likely reflects greater dietary diversity in carnivorans. While there may well be commonalities to the relationship between modularity and morphological disparity across mammalian clades, there are also significant differences related to selection pressures, ecology, and even life history. Preliminary analyses of marsupial crania, for example, display a markedly different pattern of module disparity that that reported here for placentals. Future analyses of modularity's influence on morphological evolution should continue to test the relationship between modularity and disparity to establish whether the patterns shared by Primates and Carnivora can be applied to more distantly related clades.

Of course, a complete view of modularity should not only examine the effects of integration within modules on module disparity, but also how the parcellation of a system into modules influences the morphological evolution of the entire system. To address that question, two approaches are possible. An empirical approach would require two or more clades, each with markedly different patterns of modularity in homologous systems, to compare morphological disparities across the entire system. For example, to address how the cranial modules that have been identified in placentals influence the evolution of the entire skull, one would need to compare other large clades with different patterns of cranial modularity, including different numbers of modules. Unfortunately, such an empirical approach is not possible with the three extant mammal clades because Marsupialia and Monotremata do not fulfill those requirements. Marsupials have the same general pattern of cranial modularity as placentals [32], and monotremes, although having very different patterns of cranial modularity than those observed in therian, are not diverse enough for statistically significant analyses. Diapsids are likely too long diverged from mammals to provide a meaningful comparison, but may represent a promising course for future analyses.

The topic of modularity has proven to be a rich source of novel ideas on the evolutionary process. Studies of how organisms are organized, and how this organization can dictate the course of evolution, have revolutionized evolutionary biology and provided an unexpectedly successful way of bridging scales of evolutionary study, from genetics to development to macroevolution. In this study, we have attempted to make one small step towards testing one of the most provocative hypotheses concerning the modular design of organisms: that modularity increases evolvability. If strong relationships among parts of an organism limit their variation, then breaking those relationships is crucial to maintaining the ability of organisms to vary and evolve with changing circumstances over time. However, strong relationships among functionally or developmentally-linked parts are also essential to maintain. These relationships are known to change, although they are relatively conserved across large clades [32], [49], [52]. The evolutionary significance of these changing relationships has been the subject of a great deal of theoretical modeling and discussion. However, there has thus far been no sufficiently large-scale, comparative data on both trait covariation and disparity to assess the significance of modularity in a macroevolutionary framework.

This study represents only a first pass at the question, and the models we assess here are the simplest possible, providing a starting point for more complicated scenarios and a basic methodology for testing those with empirical data. Because of the limitations discussed in detail above, we were unable to test how changes in modularity influence disparity. However, we were able to test how differences in integration influence morphological disparity, and our results show that, for the most part, there is no simple relationship between integration and disparity. Nonetheless, the results that were significant tended to support the constraint hypothesis, that integration limits variation, and thus morphological evolution, within modules.

The majority of results show that there is no simple rule or straightforward model for the influence of modularity on morphological evolution. In some cases, high integration within modules may well promote changes across the entire module, rather than impeding any change. These effects are not mutually exclusive, and high integration may well limit one kind of change while facilitating another. However, it is possible that one effect dominates over the course of evolutionary history, and empirical data is essential to determining if that is in fact the case, or if both are equally balanced over time. This study suggests that, while the total number of significant results is relatively limited, high integration constrains morphological evolution more often than it facilitates it. If this result is supported in future studies with empirical data, then it would support the idea that increasing modularity, specifically the parcellation of units into smaller subunits, may well promote evolvability by circumventing the restrictions on trait variation imposed by high integration. By breaking the bonds between traits, increasing modularity frees traits to vary independently of each other, potentially resulting in higher morphological variation and, ultimately, greater morphological evolution.

In conclusion, the results of the current analysis of within-module disparity suggested that strong integration of landmarks has little influence on morphological evolution across large time scales. However, where significant results were found, the primary effect of strong integration of landmarks was to constrain morphological variation and thus morphological evolution in the placental mammal cranium. This result was supported by separate analysis of two long-diverged orders, suggesting that it is likely to be a general pattern for placental mammals. Analyses incorporating other placental mammalian clades, particularly the more basal clades Afrotheria and Xenarthra, are necessary to establish its applicability to all placental mammals. As noted above, preliminary analysis of marsupials suggests a different pattern of cranial disparity, perhaps related to the markedly different reproductive strategies of the two therian clades.

Although most studies of modularity discuss its potential influence on morphological evolution (most intriguingly by increasing “evolvability”), there have been very few empirical studies of this effect, particularly at the macroevolutionary scale. Most studies of modularity to date have focused on bridging genetics, development, and morphology, which has provided a solid foundation for broad-scale studies and produced many provocative hypotheses on modularity's evolutionary significance. With large comparative morphometric studies increasingly possible through improvements in computing and imaging, it is hoped that more studies will focus on testing modularity's influence on morphological evolution. The study presented here provides a first step in this promising direction of research.

## Materials and Methods

### Specimens

Data were gathered from 141 specimens, representing 77 species (Table S1). Most species were represented by one male and one female specimen. Two mammalian clades, Primates (38 species) and Carnivora (39 species), were examined [53]. These two clades likely diverged in the Cretaceous period, somewhere between 100 to 65 million years ago [54]. These two clades were chosen because they have substantial morphological and ecological breadth and because they have been the focus of previous analyses of modularity and integration. These properties allowed for identification and potential isolation of confounding factors such as diet and the potential for bridging this macroevolutionary study with the many microevolutionary studies that have previously been conducted, particularly within Primates [9], [11]–[13], [21], [49], [52], [55]–[63]. The good fossil record of Carnivora also promises future analyses that include extinct taxa.

### Data Analysis

Geometric morphometric analysis of 41 3-D landmarks (Table S2, Fig. 1) was used to measure cranial disparity across 77 placental mammal species. Landmark data were collected with an Immersion Microsribe G2X digitizer, which has a reported accuracy of 0.23mm and a measured error of 0.03 mm. Only cranial landmarks of definite homology across all taxa (e.g., tripartite sutures) were used in analyses.

To measure disparity, all specimens were first aligned with generalized least squares Procrustes superimposition [64], using only the landmarks for each individual module separately, which removes differences due to rotation, translation, and scale. Partial Procrustes distance, the square root of the summed squared Euclidean distances between homologous landmarks between each specimen and the sample average shape [65], was calculated of all of the specimens. Total module disparity was defined as the sum of the partial Procrustes distances for all specimens for only the landmarks within each module.

Because modules had different numbers of landmarks (Table 1), their raw disparities could not be meaningfully compared. Configurations containing different numbers of landmarks have different measurement scales because Procrustes superimposition scales each configuration to unit centroid size (i.e., the square-root of the sum of squared Euclidean distances from each landmark to the centroid is set to 1.0) regardless of how many landmarks there are. Thus, Procrustes superimposition forces the scatter around a single landmark to progressively smaller scales when there are more landmarks, reducing the apparent magnitude of the variance at that landmark in the process. To compensate we used a randomization test to compare the observed disparity in a module with the distribution of random configurations of the same number of landmarks, with landmarks pulled from the full set, including landmarks from both “strong” and “weak” modules (Disparity A). A module was considered to have significantly high disparity if the disparity was greater than 99% of the values generated from the random distribution, but it was considered to have significantly low disparity if it was lower than 99% of the values generated from the random distribution. We also report results at the 95% level as marginally significant. Two-tailed tests for significance were used for all comparisons. 10,000 sets of *n* landmarks were drawn from the full dataset and aligned with GLS Procrustes superimposition, where *n* is the number of landmarks in the module being tested. The disparities were calculated for each of the 10,000 configurations, as described above. Analyses were also conducted in which “strong” modules (anterior oral-nasal, molar, and basicranium) were compared to disparities generated from random sets of landmarks pulled only from the “weak” modules (orbit, zygomatic-pterygoid, and vault), and vice versa (Disparity B). Similarly to Disparity A, the constraint hypothesis would predict that strong modules would have significantly lower disparities than observed in random sets of landmarks from weak modules, and that weak modules would have significantly higher disparities than observed in random sets of landmarks from strong modules. Analyses were conducted separately for Primates and Carnivora.

Additionally, a single Procrustes superimposition of all landmarks was conducted, after which landmarks were divided into the six modules. Sample variance was calculated for each landmark, and differences in landmark variances were compared across modules with a non-parametric Kruskal-Wallis test. Pairwise comparisons of modules were also conducted with non-parametric Mann-Whitney tests, with a Bonferroni correction used to account for multiple comparisons. Lastly, all landmarks were grouped into two bins, based on membership in a “strong” module or a “weak” module, and a Mann-Whitney test was used to test for differences in landmark variances between the two bins.

## Supporting Information

Table S1 List of species used in analyses.
(0.08 MB DOC)

Table S2 Landmarks used in analyses and module affiliations. ACR, acronyms for landmarks, shown in Figure 1. Modules: AON, anterior-oral-nasal; MR, molar; ORB, orbit; ZP, zygomatic-pterygoid; CV, cranial vault; BS, basicranium. S indicated symmetrical landmark measured on both right and left sides.
(0.04 MB DOC)
