# Supplementary material for: The Influence of Modularity on Cranial Morphological Disparity in Carnivora and Primates (Mammalia)
Source: PLoS One. 2010 Mar 3;5(3):e9517. doi: 10.1371/journal.pone.0009517 (PMC2831076; doi:10.1371/journal.pone.0009517)
Supplement: Table S1 — List of species used in analyses. (0.08 MB DOC) [file pone.0009517.s001.doc]

**Supplementary Table 1. List of species used in analyses.**

| **Order** | **Family** | **Species** |
| --- | --- | --- |
| Carnivora | Canidae | *Canis lupus* |
|  |  | *Canis dirus* |
|  |  | *Cerdocyon thous* |
|  |  | *Otocyon megalotis* |
|  |  | *Vulpes vulpes* |
|  | Ursidae | *Ursus americanus* |
|  |  | *Melursus ursinus* |
|  |  | *Tremarctos ornatus* |
|  |  | *Ailuropoda melanoleuca* |
|  | Ailuridae | *Ailurus fulgens* |
|  | Procyonidae | *Procyon lotor* |
|  |  | *Procyon cancrivorus* |
|  |  | *Nasua nasua* |
|  |  | *Potos flavus* |
|  | Mephitidae | *Mephitis mephitis* |
|  |  | *Spilogale putorius* |
|  | Mustelidae | *Taxidea taxus* |
|  |  | *Martes pennanti* |
|  |  | *Gulo gulo* |
|  |  | *Meles meles* |
|  |  | *Melogale personata* |
|  | Nandiniidae | *Nandinia binotata* |
|  | Viverridae | *Paradoxurus hermaphoditus* |
|  |  | *Genetta genetta* |
|  |  | *Civettictis civetta* |
|  | Felidae | *Acinonyx jubatus* |
|  |  | *Lynx rufus* |
|  |  | *Felis viverrina* |
|  |  | *Felis bengalensis* |
|  | Herpestidae | *Cynictis penicillinatus* |
|  |  | *Herpestes ichneumon* |
|  |  | *Ichneumia albicauda* |
|  |  | *Galidia elegans* |
|  | Eupleridae | *Cryptoprocta ferox* |
|  |  | *Eupleres goudoti* |
|  |  | *Fossa fossana* |
|  |  | *Galidia elegans* |
|  | Hyaenidae | *Proteles cristatus* |
|  |  | *Crocuta crocuta* |
| Primates | Lemuridae | *Eulemur fulvus* |
|  |  | *Eulemur catta* |
|  |  | *Hapalemur griseus* |
|  |  | *Varecia variegata* |
|  | Megaladapidae | *Lepilemur microdon* |
|  | Indriidae | *Indri indri* |
|  |  | *Avahi laniger* |
|  | Lorisidae | *Nycticebus coucang* |
|  |  | *Loris tardigradus* |
|  |  | *Perodicticus potto* |
|  | Galagidae | *Otolemur crassicaudatus* |
|  | Tarsiidae | *Tarsius syrichta* |
|  | Atelidae | *Alouatta seniculus* |
|  |  | *Ateles paniscus* |
|  |  | *Lagothrix lagotricha* |
|  | Pitheciidae | *Cacajao calvus* |
|  |  | *Chiropotes satanas* |
|  |  | *Pithecia pithecia* |
|  |  | *Callicebus molocha* |
|  | Cebidae | *Aotus trivurgatus* |
|  |  | *Cebus apella* |
|  |  | *Samiri sciureus* |
|  |  | *Saguinus fuscicollis* |
|  |  | *Callithrix jacchus* |
|  |  | *Cebuella pygmaea* |
|  |  | *Callimico goeldii* |
|  | Cercopithecidae | *Miopithecus talapoin* |
|  |  | *Cercopithecus aethiops* |
|  |  | *Cercocebus albigena* |
|  |  | *Macaca fascicularis* |
|  |  | *Papio anubis* |
|  |  | *Presbytis phayrei* |
|  |  | *Nasalis larvatus* |
|  |  | *Colobus rufomitratus* |
|  | Hylobatidae | *Hylobates muelleri* |
|  | Hominidae | *Pongo pygmaeus* |
|  |  | *Pan troglodytes* |
|  |  | *Gorilla gorilla* |
