# Supplementary material for: The Influence of Modularity on Cranial Morphological Disparity in Carnivora and Primates (Mammalia)
Source: PLoS One. 2010 Mar 3;5(3):e9517. doi: 10.1371/journal.pone.0009517 (PMC2831076; doi:10.1371/journal.pone.0009517)
Supplement: Table S2 — Landmarks used in analyses and module affiliations. ACR, acronyms for landmarks, shown in Figure 1. Modules: AON, anterior-oral-nasal; MR, molar; ORB, orbit; ZP, zygomatic-pterygoid; CV, cranial vault; BS, basicranium. S indicated symmetrical landmark measured on both right and left sides. (0.04 MB DOC) [file pone.0009517.s002.doc]

**Supplementary Table 2.**  **Landmarks used in analyses and module affiliations**.

| Landmarks | ACR | Module |
| --- | --- | --- |
| Premaxilla –Maxilla ventral midline suture | PM | AON |
| Premaxilla – Maxilla anterior suture (S) | Pa | AON |
| Nasals anterior midline suture | Nant | AON |
| Nasal – Premaxilla anterior suture (S) | NP | AON |
| Canine lateral extreme (S) | Cl | AON |
| Canine mesial extreme (S) | Cm | AON |
| Molar 1 anterior lateral extreme (S) | M1l | MR |
| Molar row length (lateral posterior molar) (S) | MRL | MR |
| Molar 1 anterior mesial extreme | M1m | MR |
| Jugal – Maxilla ventral suture | JMv | MR |
| Nasal – Frontal midline suture | NF | ORB |
| Jugal – Maxilla dorsal suture (S) | JMd | ORB |
| Lacrimal – Frontal – Maxilla suture (S) | LFM | ORB |
| Bulla anterior mesial extreme (S) | Ba | ZP |
| Lacrimal – Palatine – Frontal suture (S) | LPF | ZP |
| Orbitosphenoid – Alisphenoid – Frontal suture (S) | OAF | ZP |
| Jugal – Squamosal ventral suture (S) | JSV | ZP |
| Bulla - posterior lateral extreme (S) | BSL | CB |
| Occipital condyle lateral extreme (S) | OC | CB |
| Basisphenoid-Basioccipital suture (S) | BB | CB |
| Parietal – Frontal midline suture | PF | CV |
| Parietal – Occipital midline suture | PO | CV |
| Parietal – Squamosal – Alisphenoid suture (S) | PSA | CV |
| Parietal – Frontal – Alisphenoid suture (S) | PFA | CV |

ACR, acronyms for landmarks, shown in Figure 1. Modules: AON, anterior-oral-nasal; MR, molar; ORB, orbit; ZP, zygomatic-pterygoid; CV, cranial vault; BS, basicranium. S indicated symmetrical landmark measured on both right and left sides.
